# Recent Development of Non-Peptide GnRH Antagonists

**DOI:** 10.3390/molecules22122188

**Published:** 2017-12-09

**Authors:** Feng-Ling Tukun, Dag Erlend Olberg, Patrick J. Riss, Ira Haraldsen, Anita Kaass, Jo Klaveness

**Affiliations:** 1 School of Pharmacy, University of Oslo, 0316 Oslo, Norway; f.l.tukun@kjemi.uio.no (F.-L.T.); dageol@farmasi.uio.no (D.E.O.); 2 Norsk Medisinsk Syklotronsenter AS, Postboks 4950 Nydalen, 0424 Oslo, Norway; patrick.riss@kjemi.uio.no; 3 Realomics SFI, Department of Chemistry, University of Oslo, 0316 Oslo, Norway; 4 Department of neuropsychiatry and psychosomatic medicine, Oslo University Hospital, 4950 Oslo, Norway; i.h.haraldsen@medisin.uio.no; 5 Betanien Hospital, 3722 Skien, Norway; Anita.Kass@betanienhospital.no

**Keywords:** GnRH receptor, non-peptide GnRH antagonist

## Abstract

The decapeptide gonadotropin-releasing hormone, also referred to as luteinizing hormone-releasing hormone with the sequence (pGlu-His-Trp-Ser-Tyr-Gly-Leu-Arg-Pro-Gly-NH_2_) plays an important role in regulating the reproductive system. It stimulates differential release of the gonadotropins FSH and LH from pituitary tissue. To date, treatment of hormone-dependent diseases targeting the GnRH receptor, including peptide GnRH agonist and antagonists are now available on the market. The inherited issues associate with peptide agonists and antagonists have however, led to significant interest in developing orally active, small molecule, non-peptide antagonists. In this review, we will summarize all developed small molecule GnRH antagonists along with the most recent clinical data and therapeutic applications.

## 1. Introduction

Gonadotropin-releasing hormone (GnRH) or luteinizing hormone releasing hormone (LHRH) is a central regulator of the reproductive system in humans. It was first isolated from mammalian hypothalamic tissue as a linear decapeptide (pGlu-His-Trp-Ser-Tyr-Gly-Leu-Arg-Pro-Gly-NH_2_) by Scally and colleagues [1]. The GnRH peptide is produced in hypothalamic neurons and released in a pulsed fashion into the portal blood-stream supplying the pituitary gland to stimulate the synthesis and secretion of gonadotropic hormones, luteinizing hormone (LH) and follicle-stimulating hormone (FSH). The gonadotropins consecutively act on the gonads to trigger the gametogenesis, and ultimately the synthesis and release of steroidal sex hormones in both male and female [2]. Gonadal steroids in turn regulate the GnRH secretion through a positive and negative feedback and consequently surge or inhibition. Given that GnRH is playing such a significant evolutionary role in various species’ reproduction, disturbance of GnRHs signaling has therefore been implied in causing a wide spectrum of diseases; i.e., reproductive hormonal dependent oncological and neurodegenerative diseases. Therefore, treatments targeting the GnRH receptor with GnRH or its agonist analogues have attracted considerable scientific interest and lead to great commercial success [3,4]. Through down-regulation, administration of GnRH agonists lead to temporary extinction of gonadotropin secretion and sex steroidogenesis and observed chemical castration. This has been therapeutically exploited in treating hormone dependent tumors, such as prostate and breast tumors [5,6,7] to inhibit their growth. Up to now, several peptide GnRH agonists including goserelin, triptorelin and leuprolide have been approved by the FDA and reached the market [8,9]. Despite GnRH agonists’ therapeutic applications, several major clinically relevant disadvantages have emerged [5,10]. Firstly, the initial surge of gonadotropin secretion (the so-called ‘flare’ effect) before the onset of desensitization can lead to an exacerbation of the symptoms in patients through elevated sex-hormone production prior to depletion of hormone secretion. Secondly, there is a risk of bone loss from long-term administration of agonists. Thirdly, parental administration of the peptide agonists is required, due to limited bioavailability of oral formulations. In contrast to agonists, GnRH antagonists result in an immediate and reversible suppression of LH and FSH secretion without evoking the initial hyper-secretion of gonadotropins [11]. Recent clinical studies have further demonstrated the ability of GnRH antagonists to suppress gonadotropins from the onset of administration [12]. However, the inherent issues of peptidic drugs targeting GnRH-R, such as low aqueous solubility and the needs for parenteral administration have limited their clinical exploitation [13,14]. Today, there are no non-peptide GnRH antagonists in clinical use in Europe. 

Development of an orally bioavailable non-peptide GnRH receptor antagonist is therefore highly desirable, not only to address the drawbacks of peptidic GnRH receptor agonists but to ensure better patient compliance. In 1989, Abbott Laboratories reported the very first non-peptide GnRH antagonist as an antifungal drug—ketoconazole as a weak antagonist for rat GnRH receptor [15]. Since then, intensive research has been undertaken worldwide. To date, numerous small molecule GnRH antagonists representing various pharmacophores have been reported in the literature following the initial report and with a handful of chemical entities reached clinical developments (Table 1) [16].

Research activity directed towards discovery of novel non-peptidic GnRH antagonists remain high and recent reviews on this topic being published nearly one decade ago [17,18,19,20,21]. We now wish to review the developments in the field from the very first non-peptide antagonist developments, indications and the most recent available clinical data. 

## 2. Small Molecule GnRH Receptor Antagonist

### 2.1. Thieno[2,3-b]pyridine-4-one and Thieno[2,3-b]pyrimidine-2,4-one Derivatives

Based on the ‘direct screening’ of in-house chemical libraries, researchers at Takeda Chemical Industries in Japan reported the first orally-active nonpeptidic GnRH receptor antagonist for human GnRH receptor in 1998 [22]. The disclosed thiophene-based bicyclic scaffold with crucial substituents on position 2, 7, 5 and 3 was designed to mimic the (Tyr^5^-Gly^6^-Leu^7^-Arg^8^) β-turn of GnRH, a dominant structure responsible for receptor binding. Structure-activity studies (SARs) led to identification of T-98475 (compound **1**, Table 1), a thieno [2,3-*b*]pyridine-4-one analogue, which demonstrated high potency to inhibit GnRH-activation of cloned human receptor (IC_50_ = 0.2 nM). Nevertheless, species dependent selectivity is in fact is a common pattern for several classes of non-peptide GnRH-R antagonists. Compound **1** was investigated in terms of in vivo antagonism and was found to suppress plasma level of LH in Cynomolgus monkeys in a time-dependent manner after oral administration. Two different strategies were subsequently employed looking for potent GnRH antagonists with improved in vivo efficacy, and these strategies were (1) further optimization on each substituent of compound **1** and (2) replace the existing theinopyridin-4-one scaffold with other heterocyclic isosteres. The former strategy eventually led to the identification of compound **2** [23], which displayed subnanomolar in vitro activity and an improved in vivo efficacy in castrated monkeys. A more effective and sustained LH suppression (greater than 24 h) at low doses (10 and 30 mg/kg) was also observed after oral admission of compound **2**. The latter strategy looking for alternative bicyclic scaffold had resulted in a new series of molecules based on thieno[2,3-*d*]pyrimidine-2,4-diones, exemplified by TAK-013 and TAK-385 (Table 2; compounds **3** and **4**, respectively) [24,25]. Compound **3** showed high potency at both human and monkey GnRH-R receptor, with IC_50_ values of 0.1 and 0.6 nM [24]. Moreover, it also demonstrated comparable in vivo efficacy to **2** in castrated monkey. The improved oral bioavailability of compound **3** was attributed to the introduction of the terminal methoxyureido moiety at the R3-position. Through molecular modeling studies, formation of an intramolecular hydrogen bond between the aniline NH and the methoxy oxygen atom was suggested, and it was believed to shield the hydrogen bonding moieties from the solvent and therefore resulted in improvement of lipophilicity/membrane permeability [24]. Further chemical modification of compound **3** (at R_1_ and R_2_) led to the discovery of compound **4**, a highly potent and orally active GnRH antagonist [25]. In castrated monkeys, compound **4** exhibited a suppressive effect on plasma LH levels at a dose as low as 1 mg/kg for more than 24 h. Based on the biochemical and pharmacological results, both compounds **3** and **4** have been selected as candidates for clinical trials for treating sex-hormone-dependent diseases. Parallel to the literature publications, Takeda has continuously filed claims on various thiophene-based bicyclic compounds and their application as human GnRH receptor antagonists for treating hormone-dependent diseases since mid-90s. Potent analogues, such as compounds **3** and **4** were filed for protections in their preparations, indications and formulations [26,27,28,29,30]. 

### 2.2. Pyrrolo[1,2-a]Pyrimidin-7-one Derivatives

Attempts to look for new scaffolds of non-peptidic antagonists based on compound **1** (T-98475) successfully led to the discovery of pyrrolo[1,2-*a*]pyrimidines (Table 3) by Neurocrine Bioscience in the USA [31,32,33]. Initial SAR studies revealed that all non-basic compounds of this series were inactive. Combination of a 2-fluorobenzyl group at position 4 and a hydrophobic aromatic ring with an additional hydrogen bond acceptor at R_2_ position proved to improve the binding activity significantly. Subsequent optimization of aromatic ring at R_3_ demonstrated that introduction of a lipophilic isobutoxy group on the *para* position further increased potency and also led to the discovery of compound **5** [31]. Continuous modifications of this series resulted in compound **6**, which differs from **5** in removing a cyano substitute at the 3-position and introduction of an amide functionality at R_3_ [32]. Despite its high affinity, preliminary studies with compound **6** and its closed analogues revealed a liability under acidic conditions. This degradation resulted from an acid catalyzed retro-Mannich reaction on the basic side-chain at 1-position. It was reasoned that the presence of an electron withdrawing group at 3-position, such as the cyano group at compound **5**, would reduce electron density in the bicyclic system and thus helped in maintaining stability toward acid. With this rationale, Tucci et al. went ahead and introduced a fluoride at position 3 [33]. As expected, compound **7** *(K_i_* = 9 nM) was not only stable toward acid, but also maintained high affinity toward human GnRH receptor. Moreover, the electron deficient aromatic ring at position 4 was believed to be of great importance, as it interacted with electron-rich tyrosine residues in the receptor, either via π-stacking or edge-to-face interactions and led to higher binding affinity. In addition, pyrrolopyrimidones, such as compounds exemplified in Table 3 were also patented by Neurocrine Biosciences as GnRH receptor antagonists in 2002 [34]. 

### 2.3. Imidazolo[1,2-a]pyrimidin-5-one Derivatives

In 2002, parallel to the identification of pyrrolo[1,2-*a*]pyrimidines, Takeda and Neurocrine Biosciences both introduced bicyclic imidazolopyrimidinone scaffolds as new class of non-peptidic antagonists for human GnRH receptor (Table 4). As seen from compounds **8**–**12**, the imidazolo[1,2-*a*]pyrimidin-5-one analogues retain the heterocyclic 5,6-ring system but substitute the pyrrolo ring with an imidazole moiety. Compound **8**, which was identified by Takeda, was not only a potent antagonist in vitro but it also exhibited comparable potency to T-98475 (compound **1**). Based on these positive results, a heterocyclic 5,6-fused ring system bearing a phenyl group on the five-membered ring was postulated to be a key structural feature for small molecular GnRH antagonist scaffolds [35]. In addition, patent application featuring compound **8** as potential therapeutic agent for hormone-dependent disease was disclosed by Takeda prior to the literature publication [36]. 

Simultaneously, Neurocrine Biosciences also disclosed a series of imidazolo[1,2-*a*]pyrimidines as potent GnRH receptor antagonists in a patent publication, where the preparations and pharmaceutical composition were also described [37]. The high potency of imidazopyrimidines, exemplified by compounds **9**–**12** in Table 4, was attributed the existence of both the basic tertiary amine and the adjacent pyridine ring at the 3-position of the bicyclic system [38,39]. The tertiary amine, confirmed by modeling, initiated an interaction between ligand and an acidic residue within putative helical domains while the pyridine ring itself provided aromatic π-π interaction with a phenyl residue on the receptor [40]. Subsequent modifications focused on the bulky ester substituents at the 6-position, as the ester groups are prone to hydrolysis in vivo. Gross et al. reasoned that by substituting the ester group functioning as a lipophilic group and a hydrogen bond acceptor, with an arene bearing one or more hydrogen bound acceptors could circumvent the acid-liability issue. Different ring systems, such as methylenedioxyphenyl and 3-methoxyphenyl were then introduced. Combination of the new ring systems at R_1_ together with the 3-methoxyphenyl substituent at R_3_ resulted in potent compounds **10** and **11** with *K_i_* value at 11 and 4.6 nM, respectively [39]. Later SAR studies revealed that once the ester group at R_1_ was substituted with a 3-methoxyphenyl, the importance of a para-substituted aromatic ring at R_3_ in binding was somehow diminished. Evidenced by compound **12**, a highly potent molecule (*K_i_* = 5.2 nM), which had a *tert*-butyl moiety incorporated at R_3_ position [40]. 

### 2.4. Uracil Derivatives

The SAR results from the bicyclic imidazolopyrimidinones led to the discovery of a potent GnRH receptor antagonist with reduced molecular weight (compound **12**; M.W. = 553.6). Based on this, Zhu et al. further postulated that the five-membered ring in the bicyclic scaffolds might not be necessary for receptor binding [41]. This hypothesis successfully led to discovery of a novel series of monocyclic uracil derivatives as potential GnRH receptor antagonists (Table 5). 

**Scheme 1:**
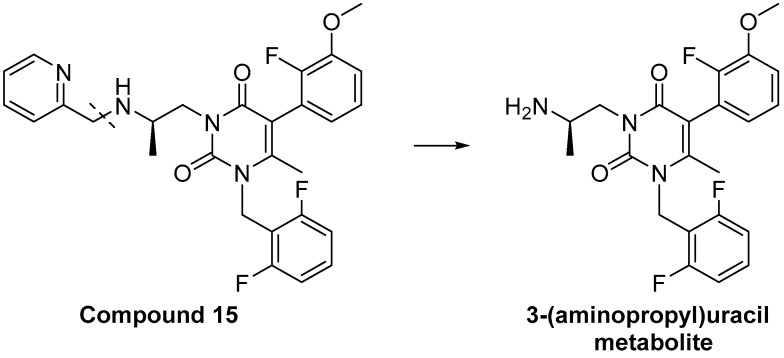
De-alkylation of compound **15** in liver microsome incubation.

Compound **13**, the first uracil GnRH-R antagonist with reasonable bonding affinity, maintained the substituents from the previous bicyclic analogues, was disclosed to have affinity at *K_i_* value of 34 nM. In the same study, Zhu et al. also reported the positive effect 6-methyl group has on the binding affinity. They reasoned that the methyl group at 6-position forced the 5-phenyl ring into a perpendicular conformation with respect to the uracil core, which in turn, may be preferred for inducing a π-π interaction with the GnRH receptor. Removing the 6-methyl group or elongating the alkyl-spacer was reported to reduce the binding affinity noticeably. In addition, due to its high lipophilicity and poor metabolic stability, compound **13** had very poor oral bioavailability (1.6%) [40]. Continuous modification included incorporating a *R*-configured methyl substituent at the β-position from N-3 of the uracil scaffold, which was believed to serve the purpose of restricting the flexible alkylamino side chain from N-3 and therefore enhance the potency. The resulting analogue **14** was seven times more potent (*K_i_* = 5.2 nM). Notably, compound **14** was found to be 90-fold more potent than its corresponding S-analogue. The phenomenon was explained by a receptor model study, in which the (*R*)-methyl group orients the pyridyl side chain toward the aspartic acid 302 on helix 7, where it was believed to improve its recognition by the receptor and therefore allowing for optimal binding [42]. Armed with these rationales, several modifications were made to further optimize the uracil compounds. First, chain length between the basic nitrogen and the 2-pyridine group at R_2_ was reduced from ethylene to methylene. Second, a fluoride was introduced at ortho position of 3-methoxy phenyl group at R_1_. Combined these changes, compound **15** was prepared with good potency against human GnRH receptor [43]. Although been metabolically more stable than compounds **13** and **14**, compound **15** still demonstrated poor oral bioavailability in rodents. In in vitro liver microsomes assay, 3-(aminopropyl)uracil, a *N*-dealkylation product was identified as the major metabolite as shown in Scheme 1.

To overcome this major obstacle, scientists in Neurocrine Biosciences decided to re-examine the SAR studies. They envisioned introducing a lipophilic group to a neighboring carbon of the basic nitrogen at R_2_ would circumvent the issue (Compound **16**). Compounds **16** was not only a potent human GnRH-R antagonist, but it also exhibited good oral bioavailability in both mice and monkeys (42% and 22%, respectively) [44]. Despite the improved oral bioavailability profile, the half-life of compound **16** was still too short to qualify for the desirable pharmacokinetic properties. Continuous optimizations of the uracil series subsequently led to the discovery of compound **17**, also known as NBI 42902, with significant improvements in both antagonist activity (*K_i_* = 0.56 nM) and pharmacokinetic properties. While the potency of compound **17** remained high at monkey GnRH receptor (*K_i_* = 3.5 nM), the affinity at the rat receptor were negligible (*K_i_* = 3000 nM). Resembling species dependent selectivity as common observed for small non-peptide GnRH-R antagonists. Despite that, compound **17** had been developed into clinical evaluation for potential treatment of GnRH related diseases [45], the existence of atropisomers (rotational diastereoisomers) posed potential difficulties in ensuring reproducibility in manufacturing. The methyl group at 6-position was believed to cause this phenomenon. Despite being a crucial feature of the uracil-pharmacophore series, the 6-methyl group was postulated to hinder carbon-carbon bond rotation between the 2-fluroro-3-methoxyphenyl ring and the uracil ring. This in turn led to observation of *R*- and *S*-rotamers at room temperature [46]. In the efforts to avoid the atropisomerism (rotational diastereoisomers), several modifications were produced. Firstly, the 2-fluoro-3-methoxyphenyl group was substituted with various heteroaromatic groups. Compound **18**, a 5-chloro-2-thienyl analogue, was reported to be most potent [47]. However, no further publication regarding this class of derivatives has been reported since. Secondly, the methyl group at 6-position was removed [48]. A table of potent uracil derivatives without 6-methyl group were then prepared (compounds **19**–**21**). As postulated, atropisomeric properties were not observed [46], however, without the 6-methyl group less potent compounds were obtained (**19** vs. **17**). From receptor modeling and mutagenesis studies, the substituted benzyl group at the N-1 was proposed to bind in a pocket formed by three tyrosine residues (Tyr-282, 284 and 290) of transmembrane domain six. An electron-deficient aromatic ring at the N-1 position was therefore suggested to ensure a strong interaction with the residues. Compounds **20** and **21** were then prepared with compatible potency to compound **17** [46]. In spite of its high potency at human GnRH-R, compounds **20** was also a potent CYP3A4 inhibitor (IC_50_ = 0.1 µM), which posed potential risk of raising drug-drug interaction with a large group of chemically useful drugs [49]. By introducing a carboxylic acid moiety on the basic nitrogen group, dramatic reduction in the inhibitory activity at CYP3A4 was achieved while high GnRH antagonist potency and good oral bioavailability were maintained (zwitterions **22** and **23**) [50]. Based on these promising characteristics, compound **23**, also known as Elagolix was selected as a clinical compound for treatment of sex-hormone-dependent diseases. Parallel to the literature publications on uracil compounds, Neurocrine Biosciences has also described their preparation and pharmaceutical compositions published in a series of patents [51,52,53,54].

While being the most clinical advanced nonpeptidic GnRH antagonist to date, Elagolix appears to exhibit low oral bioavailability in both rats and monkeys (5.8% and 11%, respectively). To improve the oral absorption of the uracil scaffold, Kim et al. at SK Chemicals in South Korea decided to re-visit the structure-activity analyses [55]. After re-examining the structure-activity analysis, they concluded that R_1_ position of the uracil moiety offers the most readily opportunity for further modifications. A library of novel uracil scaffolds comprising of various nonaromatic heterocycles at N-3 were then prepared and exemplified at Table 6. Initial screening of various uracil analogues successfully led to the discovery of compounds **24** & **25**. With their promising potency, compounds **24** and **25** were employed as starting leads for further modifications. While preserving most of the functional groups at the uracil core of compound **24**, different substituents were systemically introduced onto the benzene ring in R_1_. Among various substituents, 2-F, 2-CN and 3-CF_3_ generally led to great improvements in binding potency (compounds **26**, **27** and **28**). Notably, compound **28** also exhibited comparable potency to Elagolix. From previous SAR studies of Elagolix [49], antagonistic activity was shown to improve greatly by replacing the 1-(2,6-difluoro)benzyl group at the uracil core with a 1-(2-fluoro-6-trifluoromethyl)benzyl group. Same trend was again observed here, as 1-(2-fluoro-6-trifluoromethyl)benzyl analogue (compound **29**) was more potent than its corresponding 1-(2,6-difluoro)benzyl analogue (compound **28**). While exhibiting potent binding affinity (compounds **28** and **29**) for human GnRH-R, their inhibitory effects on NFAT activation, a calcium dependent nuclear transcription factor that with its reporter gene assays provide a reliable method for monitoring the activation of GPCR signaling pathways, were relatively weak. To improve the inhibitory effect towards NFAT while maintaining the potency, Kim et al. turned their attention to the 5-(4-pyridylclicpiperazinyl) at R_1_ (compound **25**). Numerous substituents were incorporated onto the 4-pyridyl ring and rather disappointingly, none of the derivatives resulted in any improvement of the NFAT inhibition (compounds **30** and **31**). Slow dissociation kinetics, as previously reported with Elagolix, was believed to contribute to this apparent discrepancy in human GnRH-R binding affinity and NFAT inhibition. Different fused bicyclic systems and five-membered heterocyclic groups on the 4-pyridyl ring were next investigated. While the introduction of the fused ring systems on 4-pyridyl ring led to no change in activity, the incorporation of 2-furyl or 2-thiophenyl groups resulted in promising outcomes (compounds **32** and **33**). These results prompted Kim et al. to further investigate the SAR of these five-membered heterocycles. Eventually, a potent analogue (compound **34**), with an electron-withdrawing substituent at the furyl ring, was discovered. It was reported to exhibit potent activity for both binding affinity (IC_50_ = 0.95 nM) and NFAT inhibition (IC_50_ = 12.6 nM). However, it demonstrated rather strong CYP3A4 inhibition (96% at 10 µM) when subjected to screen for human CYP isozyme inhibition. 

Similar outcomes were also reported by Neurocrine Bioscience when developing Elagolix. Incorporation of a polar group, such as butyric acid on the primary amine group at R_2_ position was reported to solve the problem. SK Chemicals again confirmed the finding and corresponding analogue, compound **35**, was reported to exhibit much lower CYP3A4 inhibition. Based on those positive findings, compound **35** was then selected for further investigation. Like many small molecular GnRH antagonists, compound **35** was also found to demonstrate selective antagonistic activity among different species. Pharmacokinetic and pharmacodynamic evaluations of compound **35** in rats and monkeys revealed improved bioavailability (15.6% and 13.2%, respectively) and better gonadotropic LH suppression when in comparison with Elagolix. Given these favorable characteristics, compound **35** has been chosen for further developments.

### 2.5. Indole Derivatives

Since the mid of 1990s, Merck has reported tryptamine derivatives as potent GnRH receptor antagonists in series of patents [56,57,58,59]. Derived from the initial lead compound with micromolar binding affinity on the rat GnRH receptor (IC_50_ = 3 µM), progressive efforts in optimizing the affinity of indole structures were taken and exemplified here as compounds **36**–**41** (Table 7). 

First, introduction of a 3,4-dimethoxyphenyl group on the indole core, resulted in compounds with nanomolar potency [60]. Second, the metabolically unstable phenolic hydroxyl group at R_1_ in compound **36** was substituted with other surrogates, such as a heterocyclic 4-pyridyl ring. Compound **37** was then reported with improved potency (IC_50_ = 16 nM) [61]. Third, incorporation of substitutions at R_2_, such as functionalized carboxamides, led to increase in receptor affinity noticeably (compound **38**). In addition, a branched (gem-dimethylated) spacer inserted between the indole and the carboxamides further improved potency as it helped in minimizing potential metabolic cleavage (compound **39**) [62]. Fourth, addition of a chiral β-methyl group on the basic amide side chain and reduction of its spacer to two-carbons at R_2_ enhanced both the potency and oral bioavailability (Compound **40**, IC_50_ = 0.6 nM).In in vivo efficiency study, a single dose of compound **40** at 10 mg/kg provided complete suppressed of LH plasma levels for ˃12 h in castrated male rates (IC_50_ = 1.7 nM for rat GnRH-R) [63]. Last, oxidation of the pyridine nitrogen coupled with methyl substitution at ortho position resulted in improvements of both affinity and pharmacokinetic properties. The obtained 2-methylpyrid-4-yl-*N*-oxide **41** was reported to have greater than 20% bioavailability in both dogs and rats [64].

### 2.6. Quinolone Derivatives

In addition to the indole analogues, Merck also disclosed a library of quinolone derivatives as novel GnRH receptor antagonists in a string of patent documents [65,66,67]. The lead compound, identified after screening of in-house sample collections, was reported as having micromolar binding affinity for rat GnRH-R (IC_50_ = 10 µM). From the initial SAR study, several important structural features were recognized to be crucial for antagonist activity. These features included preservation of a chlorine atom on the quinolone ring, incorporation of a nitro group at R_2_ and introduction of an alkyl cyclic amines at R_1_. In addition, a 3-carbon spacer between the quinolone oxygen and the basic amine on R_1_ was also found to play an important role in enhancing potency (Table 8). 

Compound **42**, the first nanomolar affinity compound of this series was then prepared (IC_50_ = 32 nM) [68]. Further SAR studies demonstrated the ring size of the alkyl cyclic amine substituent at R_1_ did not have an effect on binding affinity as 4-, 5-, 6- and 7-membered amines were of similar potency. The chirality at R_1_, on the contrary, told a different story. It appeared that S-configuration was significantly more potent than its corresponding R-isomers. Compound **43**, with IC_50_ value of 10 nM, was reported to be at least fifteen-fold more potent at the rat GnRH-R than its R-counterpart [69]. Continuous efforts in improving the potency led Merck to diversify both the nitro group at R_2_ and the aryl group on the quinolone ring. Among various substituents, pyrimidine-carboxamide was shown to be the superior substituent (compound **44**) [70]. As for the aryl group at 3-position, a 3,4,5-trimethylphenyl substituent was the optimal as it further increased the affinity by threefold (compound **45**, IC_50_ = 0.3 nM) [71]. Subsequent report published by Merck also described installations of different cyclic basic amine moieties, such as azetidine at R_1_, helped in maintaining binding affinity, functional activity, and in vivo efficacy in primates (compound **46**). As commonly observed in non-peptid GnRH antagonists, the species dependent selectivity was again observed. Compound **46** was found to possess excellent affinity to cloned human GnRH receptor (IC_50_ = 0.44 nM), but much reduced binding affinity for rat and dog receptors (10- and 150-fold shifts in binding, respectively) [72]. Attempts in extending the SAR around the quinolone core successfully led to disclosure of various potent analogues containing neutral (nonbasic) side chains at R_1_. The newly discovered non-basic heterocycles, such as cyclic ethers and cyclic lactams, were effective pharmacophores for binding and functional antagonist activity on the human GnRH receptor (compounds **47** and **48**) [73].

### 2.7. Furamide Derivatives

In 2002, Pfizer disclosed another novel series of nonpeptide GnRH receptor antagonists featuring furan-2-carboxamide template in different patent documents (Table 9) [74,75]. Notably, the furan-based structures are very different from the previously mentioned scaffolds, as they do not possess a 5,6-membered heterocyclic core. The initial lead compound **49**, was identified after in-house screening and reported as a moderately potent GnRH receptor antagonist (*K_i_* = 40 nM). Despite its potency, the guanidine moiety in the structure was suspected to cause potential absorption issues and therefore, may pose a liability in future developments. Continuous SAR studies suggested that the guanidine moiety at R_2_ does not play a crucial role in overall binding affinity and could be substituted with other functionalities [76]. Further optimization at R_2_ led to substitution of the initial guanidine moiety with different “caged” guanidines, such as mono- or diaminopyrimidines. From both in vitro and in vivo studies, the diaminopyrimidines were more potent and functional active than the mono-aminopyrimidine derivatives (compounds **50** & **51**). The superior in vitro activity of compound **51** was attributed to the 2-*S*-tetrahydrofurylmethyl group, as it provided additional hydrophobic interactions with receptors. Unlike most classes of nonpeptide GnRH receptor antagonists, species dependent selectivity was not observed among furan-2-carboxamides. This permitted the use of less expensive rodent species for in vivo activity, efficacy and toxicology assessments (compound **51**, human *K_i_* = 8 nM vs. rat *K_i_* = 1.6 nM) [77]. In addition, intravenous administration of compound **51** at 20 mg/kg successfully suppressed LH level for up to 6 h. To improve the limited bioavailability, Anderes et al. in Pfizer continued to modify R^2^ and eventually reported a potent, orally active GnRH receptor antagonist, compound **52**. Subsequent in vitro and in vivo activities of compound **52** were also conducted and reported by the same team [78]. In competition binding assays, compound **52** shown no species differences and bound with comparable low nanomolar affinities to human, rat and mouse receptors (*K_i_* = 6.0, 3.8 and 2.2 nM, respectively). As for in vivo efficacy studies, oral administration of compound **52** completely suppressed LH and testosterone levels in both castrated and intact male rate, although at a relatively higher dose (100 mg/kg). Guided by these structure-activity relationships and biomarker measurements, Pfizer eventually disclosed compound **53**, the first compound of this class that exhibited picomolar-affinity at both human and rat GnRH receptors. Apart from its improved affinity profiles, compound **53** also suppressed LH and testosterone in blood levels completely for half of the dose concentration of compound **52** (50 mg/kg) [79]. In addition, compound **53** demonstrated high selectivity toward GnRH receptors and low drug-drug interaction potential with various cytochrome P450s. Recently, several novel brain imaging agents, based upon structures of small non-peptide GnRH antagonists, have also been developed in response to the increased interest in positron emission tomography (PET). Among them, compounds **54** and **55** were reported to pass the blood-brain-barrier and demonstrate high receptor affinity in vitro. Despite it failed to reveal significant specific binding in brain of living rats, it still serves as a foundation for the further development of small molecule GnRH PET ligands suitable for imagine CNS GnRH-R distribution [80,81].

### 2.8. Benzimidazole Derivatives

Bayer, in 2005 described a series of benzimidazole derivatives as a novel class of non-peptide GnRH antagonists (Table 10). Compound **56**, the lead compound which was identified after screening of in-house compound library, exhibited low micromolar potency for both human and rat receptors (IC_50_ = 3.4 and 3.5 µM, respectively). From initial SAR studies, several key functional groups were noted for ensuring high potency. First, the substitution patterns on the sulfonamide phenyl ring had strong effect on potency, with electron withdrawing para-substitute being the optimal. Second, basic functionalities, such as secondary or tertiary amines at R_4_ position of benzimidazole core also improved the affinity. Compound **57** was then synthesized and it exhibited improved affinities for both human and rat receptors [82]. Continues optimizations on the benzimidazole core led to the discovery of compound **58**, the first nanomolar GnRH inhibitor within the series. The significantly improved potency of compound **58** was attributed to the introduction of *tert*-butyl urea functionality at R_4_. The urea moiety, containing both hydrogen bond donors and acceptor, provided crucial interaction with the receptor and therefore boosted the activity. Compound **58**, however, demonstrated strong CYP3A4 inhibition [83]. Parallel efforts at Bayer looking for for new pharmacophores also prompted Tatsuta et al. to report another lead which also based on benzimidazole core (compound **59**). SAR at R_1_ and R_4_ positions were investigated and reported. Combination of several modifications, such as urea functionality at R_1_ and a substituted phenyl ring at R_4_ led to the identification of a potent candidate (compound **60**) [84]. 

### 2.9. Piperazine-Benzimidazole Derivatives

In 2008, Wyeth disclosed several patents claiming a novel series of small molecule GnRH antagonists based on benzimidazole pharmacophore (Table 11) [85,86]. The leads, compounds **61** and **62** were identified after screening of a GPCR directed library. Both compounds **61** & **62** were structurally related 4-piperazinylbenzimidazoles and had modest bindings on human GnRH receptors (IC_50_ = 1.54 and 0.8 µM, respectively) [87]. Subsequent structure optimizations, including replacement of the bicyclic structure at R_1_ with a monocyclic heterocycle and introduction of a 4-*tert*-butylphenyl group at R_2_ position, successfully led to discovery of a potent inhibitor with nanomolar affinity for human receptors (compound **63**, IC_50_ = 1.7 nM). Notably, compound **63** also exhibited good potency at the rat receptor (IC_50_ = 17.5 nM). In in vivo pharmacokinetic analysis, compound **63** demonstrated excellent bioavailability (*F* = 74%) and good serum LH suppression in rats at single dose of 30 mg/kg for at least 6 h. However, its poor solubility, strong CYP3A4 inhibition and poor liver microsome stability imposed great liabilities on future developments. Hoping to mitigate the issues through structural modification, researchers in Weyth postulated that the appended heterocycle ring at R_1_ was the key part in improving the in vitro pharmaceutical profile. Various fused heterocycles were introduced and among them, quinoxaline derivative (compound **64**) was most promising. Not only had compound **64** demonstrated potent GnRH receptor affinities (human GnRH IC_50_ = 12 and rat GnRH IC_50_ = 71 nM), but also shown improvement in in vitro pharmaceutical properties. In addition, compound **64** was reported to suppress LH levels for up to 24 h in rat (single dose of 30 mg/kg) and had bioavailability of 74% (*F*%) [88]. 

### 2.10. Spiroindoline Derivatives

Since 2014, a series of patents claiming novel spiroindoline derivatives as GnRH receptor antagonists has been disclosed by Bayer (Table 12) [89,90,91,92,93]. Compound **65**, the first reported oral active GnRH antagonist for this series, was reported to be moderately potent (IC_50_ = 104 nM) as an isolated enantiomer. Moreover, oral administration of compound **65** in ovariectomized rats led to 89% of LH suppression for 6 hours at single dose of 10 mg/Kg [89]. Continuous SAR studies based on compound **65** subsequently led to a series of GnRH antagonists such as compounds **66**–**68** with improved potency. In the patent document (WO14166958), compound **66** was also claimed to suppress LH levels for up to 8 h in rat (single dose of 10 mg/kg) and had oral bioavailability of 87% (*F*%). Noteworthily, compounds **66**–**68** were all presented her as isolated enantiomers [90]. Very recently, Bayer disclosed the further optimization of these analogues, exemplified by compound **69** as a potent GnRH receptor antagonist. Compound **69**, which had an additional electron-withdrawing substitute at R_3_, was reported to have IC_50_ value of 3.5 nM [91]. WO15082374 described a series of modification on the top of the indole (X position), which then yielded some potent GnRH antagonists such as compound **70**. Interestingly, both diastereomers of compound **70** possessed potent antagonist activities (IC_50_ = 1.7 and 1.4 nM, respectively) [92]. In their most recent patent document (WO15091315), the structure-activity relationship at both X and R_2_ positions were elucidated along with their preparation and pharmaceutical compositions. Compound **71**, with a piperidine substituent on spiroindoline core and a methyl ethylene at R_2_ position, was then disclosed as potent GnRH receptor antagonist [93].

### 2.11. Other Compounds

A group of propane-1,3-dione derivatives **72** were disclosed as GnRH antagonist by Astellas Pharma in patent documents filed in Japan in 2004. According to the patent documents the compounds have IC_50_ values as GnRH antagonists from 0.08 nM [94].

In 2013, SK Chemical in South-Korea also published another group of tricyclic urea derivatives (**73**) as GnRH antagonists. In particular, on the compound reported in the patent document (**74**) was documented to demonstrate 100% GnRH inhibition at 10 nM concentration [95].

Most recently, a group of compounds with 2-(4-*tert*-butylphenyl)-4-piperazinyl-benzimidazole scaffold were reported to possess affinity in nanomolar range and the most potent compound was described as compound (**75**) [96].

## 3. Clinical Developments of Small Molecule GnRH Receptor Antagonist

Over the last two decades, scientific research has worked toward developing orally active non-peptide GnRH antagonists, with the aim to overcome drawbacks associated with peptide GnRH analogs. Through the extensive efforts, several classes of non-peptide ligands have advanced into clinical development (Table 13). TAK-013 and TAK-385, developed by Takeda, had both entered clinical trials for the acute treatment of endometriosis and uterine fibrosis as well as assisted fertilization protocols (Table 14). While positive preclinical results were reported and Phase II trials were reportedly being conducted worldwide, the development of TAK-013 was discontinued in 2005 for strategic reasons 95 TAK-385, on the other hand, has continually advanced into Phase III trial targeting prostate cancer. For uracil series, there are two analogues that have entered clinical developments, namely NBI-42902 and Elagolix. NBI-42902 was reported to exhibit good adsorption, pharmacokinetic parameters and activity from the earlier Phase I studies [97]. Despite of the positive results and no adverse effects were reported at the time, development with NBI-42902 was discontinued after phase II trials in 2005. Elagolix, the most clinically advanced non-peptidic compound studied to date, have entered two phase III trials. From previous clinical studies, Elagolix was found to demonstrate acceptable safety and tolerability profile along with mild adverse events. Phase III trials were completed in November 2016, and reported positive safety profiles and high efficacy for Elagolix. In September 2017, AbbViefiled a New Drug Application to the FDA for Elagolix in endometriosis-associated pain. [16,98,99,100]. ASP-1707, developed by Astellas Pharma in Japan, has also entered phase II for the treatment of not only endometriosis but also of a new indication-rheumatoid arthritis. To date, structure of ASP-1707 remained unknown while its results from clinical trials were also undisclosed. SKI-2670, developed by SK Chemical Ltd. in South Korea, had entered a phase I clinical trial in 2014. Neither the structure of SKI-2670 nor the results has been disclosed [101].

## 4. Potential New Indications for Non-Peptide GnRH Antagonists

Clinical studies for both non-peptide or peptide GnRH antagonists are either in recruiting phase or have been completed with targeting to various diseases. Relugolix has entered various clinical trials targeting prostate cancer, endometriosis related pain, uterine fibroids and heavy menstrual bleeding associating with uterine fibroids [109]. Elagolix has been reported to target endometriosis associated pain, premenopausal women with heavy menstrual bleeding which is associated with uterine fibroids. In addition, efficacy and safety study of Elagolix in combination with estradiol/norethindrone acetate for the management of heavy menstrual bleeding associated with uterine fibroids in premenopausal women has also been reported [110]. Cetrorelix, a peptide GnRH antagonist, is also shown in clinical studies to produce rapid anti-inflammatory effects in rheumatoid arthritis patients with high gonadotropin levels [111]. This has motivated for further clinical studies on GnRH antagonists: “A Study to Evaluate the Pharmacokinetics of ASP1707 and Methotrexate in Patients with Rheumatoid Arthritis” and “A Study to Evaluate the Efficacy and Safety of ASP1707 in Postmenopausal Female Patients with Rheumatoid Arthritis Taking Methotrexate”. Astellas Pharma is the sponsor for both studies [112].

## 5. Conclusions

For the past decades, intensive research activity has been directed towards identifying potent orally active, non-peptide GnRH antagonist. As results, a vast collection of structurally diversified small molecules were discovered possessing high affinity and efficacy. Some of the new molecules have advanced into clinical development, but detailed reports of these trials are scarce. Nevertheless, based on the available published data, orally available, non-peptide GnRH-R antagonists may represent a novel therapeutic option for treatment of hormone-dependent diseases. It is, therefore, reasonable to anticipate that non-peptide GnRH-R antagonists will find their way into clinic in the near feature. 

## Figures and Tables

**Table 1:**
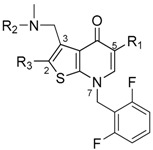
Thieno[2,3-*b*]pyridine-4-one derivatives and their binding affinities at human GnRH-R.

Compound	R_1_	R_2_	R_3_	IC_50_ (nM)
**1**	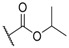	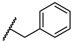	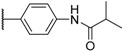	0.2
**2**	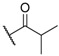	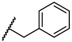	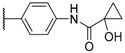	0.1

**Table 2:**
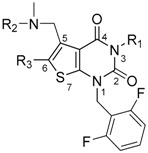
Thieno[2,3-*d*]pyrimidine-2,4-one derivatives and their binding affinity at human GnRH.

Compound	R_1_	R_2_	R_3_	IC_50_ (nM)
**3** TAK-013 Sufugolix	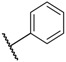	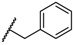	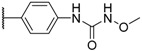	0.1
**4** TAK-385 Relugolix	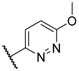	Me	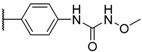	0.33

**Table 3:**
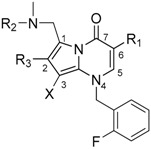
Pyrrolo[1,2-*a*]pyrimidin-7-one derivatives and their binding affinity at human GnRH-R.

Compound	R_1_	R_2_	R_3_	X	*K_i_* (nM)
**5**	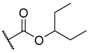	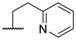	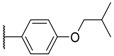	CN	25
**6**	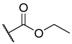	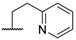	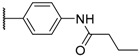	H	1.2
**7**	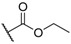	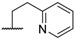	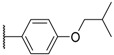	F	9

**Table 4:**
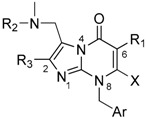
Imidazolo[1,2-*a*]pyrimidin-5-one and their binding affinity at human GnRH-R.

Compound	R_1_	R_2_	R_3_	Ar	X	*K_i_* (nM)
**8**	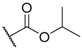	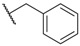	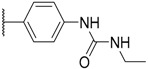	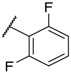	H	0.3 ^1^
**9**	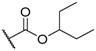	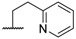	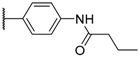	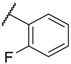	H	7.5
**10**	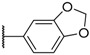	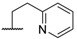	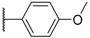	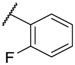	Me	11
**11**	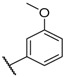	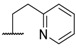	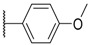	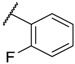	Me	4.6
**12**	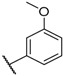	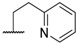	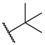	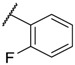	Me	5.2 ± 0.6

^1^ The binding affinity is reported as the IC_50_ value.

**Table 5:**
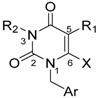
Uracil derivatives and their binding affinity at human GnRH-R.

Compound	R_1_	R_2_	Ar	X	*K_i_* (nM)
**13**	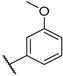	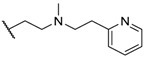	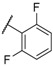	Me	34
**14**	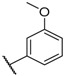	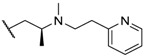	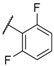	Me	5.2
**15**	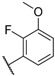	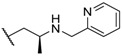	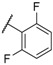	Me	1.1
**16**	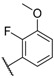	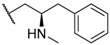	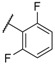	Me	8.1
**17** NBI 42902	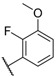	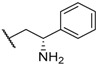	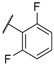	Me	0.56
**18**	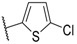	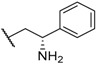	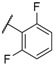	Me	2
**19**	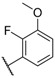	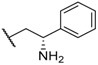	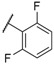	H	5.3
**20**	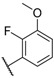	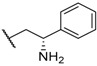	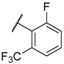	H	0.64
**21**	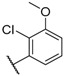	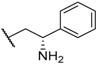	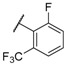	H	0.45
**22**	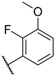	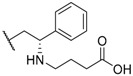	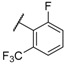	H	1.2
**23** Elagolix	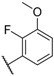	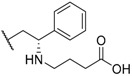	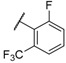	Me	0.9

**Table 6:**
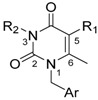
Uracil derivatives and their binding affinity at human GnRH-R.

Compound	R_1_	R_2_	Ar	IC_50_ (nM)
**24**	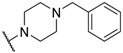	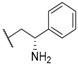	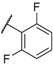	12
**25**	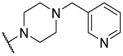	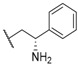	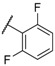	13.9
**26**	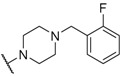	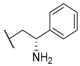	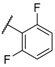	4.1
**27**	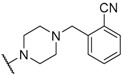	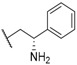	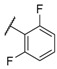	6.8
**28**	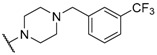	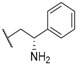	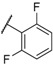	0.91
**29**	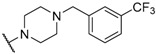	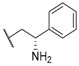	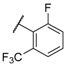	0.52
**30**	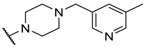	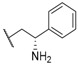	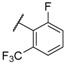	7.18
**31**	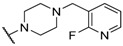	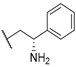	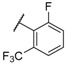	1.07
**32**	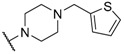	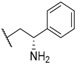	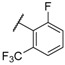	1.27
**33**	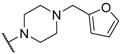	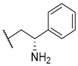	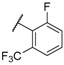	2.76
**34**	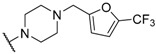	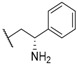	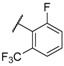	0.95
**35**	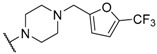	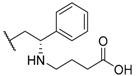	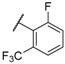	0.44

**Table 7:**
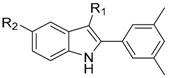
Indole derivatives and their binding affinity at human GnRH-R.

Compound	R_1_	R_2_	IC_50_ (nM)
**36**	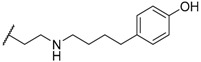	H	50
**37**	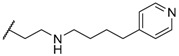	H	16
**38**	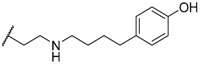	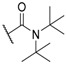	5.7
**39**	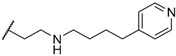	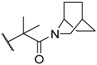	1.4
**40**	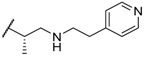	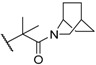	0.6
**41**	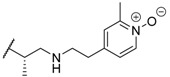	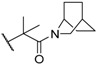	0.3

**Table 8:**
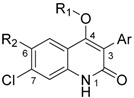
Quinolone derivatives and their binding affinity at human GnRH-R.

Compound	R_1_	R_2_	Ar	IC_50_ (nM)
**42**	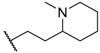	NO_2_	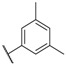	32
**43**	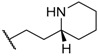	NO_2_	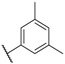	10
**44**	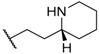	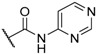	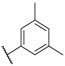	0.9
**45**	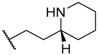	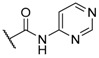	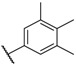	0.3
**46**	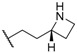	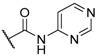	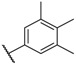	0.44
**47**	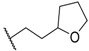	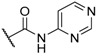	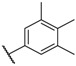	0.6
**48**	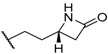	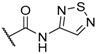	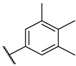	0.1

**Table 9:**
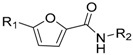
Furamide derivatives and their binding affinity at human GnRH-R.

Compound	R_1_	R_2_	*K_i_* (nM)
**49**	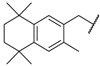	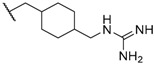	40
**50**	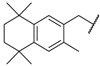	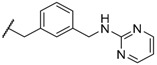	130 ± 10
**51**	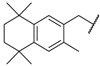	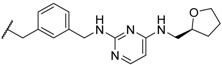	8 ± 0.8
**52** CMPD1	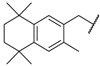	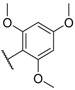	6.0 ± 0.8
**53**	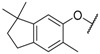	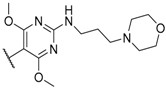	0.4
**54**	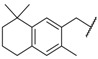	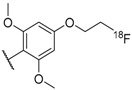	0.3
**55**	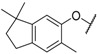	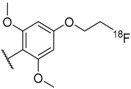	1.3

**Table 10:**
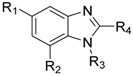
Benzimidazole derivatives and their affinity at human GnRH-R.

Compound	R_1_	R_2_	R_3_	R_4_	IC_50_ (nM)
**56**	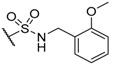	H	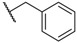	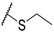	3400
**57**	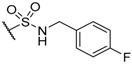	H	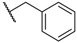	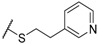	120
**58**	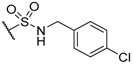	H	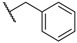	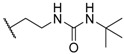	4.2
**59**	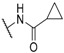	H	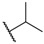	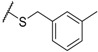	6500
**60**	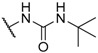	H	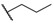	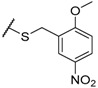	18

**Table 11:**
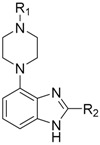
Piperazine-benzimidazole derivatives and their binding affinity at human GnRH-R.

Compound	R_1_	R_2_	IC_50_ (nM)
**61**	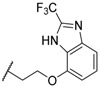	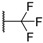	1540
**62**	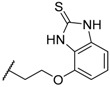	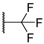	800
**63**	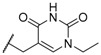	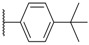	1.7 ± 0.65
**64** WAY-207024	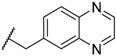	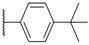	12 ± 4.2

**Table 12:**
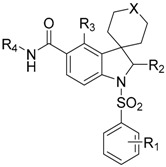
Spiroindoline derivatives and their binding affinity at human GnRH-R.

Compound	R_1_	R_2_	R_3_	R_4_	X	IC_50_ (nM)
**65**	4-F	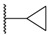	H	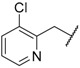	SO_2_	104
**66**	4-F	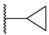	H	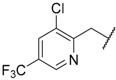	SO_2_	21
**67**	3-CN	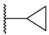	H	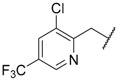	SO_2_	14
**68**	4-CN	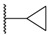	H	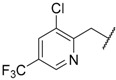	SO_2_	10
**69**	4-F	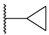	Cl	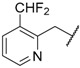	SO_2_	3.5
**70**	4-F	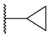	H	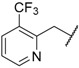	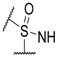	1.7
**71**	4-F	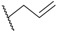	H	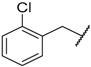	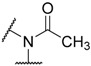	11.1

**Table 13:** Other GnRH antagonist derivatives.

Compound	Structure
**72** ^1^	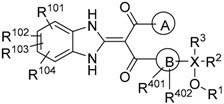
**73** ^1^	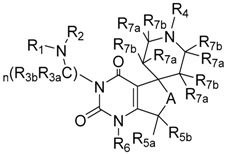
**74**	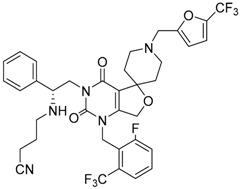
**75**	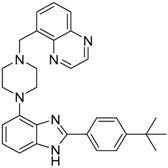

^1^ Structural details are not disclosed and is therefore represented with a general structure.

**Table 14:** Non-peptide GnRH antagonists in clinical trials.

GnRH Antagonists	Clinical Trial	Indications Evaluated in Clinical Trials	Industry	Published Human Data
TAK-013 (Sufugolix) Compound **3**	Phase II ^1^	Endometriosis, uterine fibroids	Takeda	[102]
TAK-385 (Relugolix) Compound **4**	Phase III	Prostate Cancer	Takeda	-
	Phase II	Endometriosis, uterine fibroids, prostate cancer	Takeda	[103]
NBI-42902 Compound **17**	Phase II ^1^	Hormone dependent diseases	Neurocrine Biosciences	[97]
Elagolix Compound **23**	Phase III	Endometriosis, uterine fibroids	Neurocrine Biosciences	[104,105,106,107]
ASP-1707	Phase II	Endometriosis	Astellas Pharma	[108]
SKI2670 ^2^	Phase I	Endometriosis	SK Chemical	[101]

^1^ Discontinued clinical trials; ^2^ Unknown structures.
